# Using aerobic exercise to evaluate sub-lethal tolerance of acute warming in fishes

**DOI:** 10.1242/jeb.218602

**Published:** 2020-05-07

**Authors:** Felipe R. Blasco, Andrew J. Esbaugh, Shaun S. Killen, Francisco Tadeu Rantin, Edwin W. Taylor, David J. McKenzie

**Affiliations:** 1 Department of Physiological Sciences, Federal University of São Carlos, 13565-905 São Carlos, SP, Brazil; 2 Joint Graduate Program in Physiological Sciences, Federal University of São Carlos – UFSCar/São Paulo State University, UNESP Campus Araraquara, 14801-903 Araraquara, SP, Brazil; 3 Marine Science Institute, University of Texas at Austin, Austin, TX 78373, USA; 4 Institute of Biodiversity, Animal Health & Comparative Medicine, College of Medical, Veterinary & Life Sciences, University of Glasgow, Glasgow G12 8QQ, UK; 5 School of Biosciences, University of Birmingham, Birmingham B15 2TT, UK; 6 MARBEC, Université de Montpellier, CNRS, Ifremer, IRD, 34000 Montpellier, France

**Keywords:** CT_max_, *Oreochromis niloticus*, *Piaractus mesopotamicus*

## Abstract

We investigated whether fatigue from sustained aerobic swimming provides a sub-lethal endpoint to define tolerance of acute warming in fishes, as an alternative to loss of equilibrium (LOE) during a critical thermal maximum (CT_max_) protocol. Two species were studied, Nile tilapia (*Oreochromis niloticus*) and pacu (*Piaractus mesopotamicus*). Each fish underwent an incremental swim test to determine gait transition speed (*U*_GT_), where it first engaged the unsteady anaerobic swimming mode that preceded fatigue. After suitable recovery, each fish was exercised at 85% of their own *U*_GT_ and warmed 1°C every 30 min, to identify the temperature at which they fatigued, denoted as CT_swim_. Fish were also submitted to a standard CT_max_, warming at the same rate as CT_swim_, under static conditions until LOE. All individuals fatigued in CT_swim_, at a mean temperature approximately 2°C lower than their CT_max_. Therefore, if exposed to acute warming in the wild, the ability to perform aerobic metabolic work would be constrained at temperatures significantly below those that directly threatened survival. The collapse in performance at CT_swim_ was preceded by a gait transition qualitatively indistinguishable from that during the incremental swim test. This suggests that fatigue in CT_swim_ was linked to an inability to meet the tissue oxygen demands of exercise plus warming. This is consistent with the oxygen and capacity limited thermal tolerance (OCLTT) hypothesis, regarding the mechanism underlying tolerance of warming in fishes. Overall, fatigue at CT_swim_ provides an ecologically relevant sub-lethal threshold that is more sensitive to extreme events than LOE at CT_max_.

## Abstract (precis)

**Summary:** Fatigue from sustained aerobic exercise can be used to measure sub-lethal tolerance of acute warming in fishes.

## INTRODUCTION

Aquatic habitats are undergoing increasingly rapid human-induced environmental change, especially in response to climate warming. Projections indicate a gradual increase in seasonal temperatures by up to 4°C by 2100, accompanied by an increased frequency of extreme and acute warming events at localized scales (Frölicher and Laufkötter, 2018; IPCC, 2014, 2019). This will challenge resident animals, particularly ectotherms such as fishes, whose physiology is profoundly influenced by temperature, with consequences for fitness traits such as survival, growth and reproduction (Perry et al., 2005; Pörtner and Knust, 2007; Smale et al., 2019). Therefore, it is critical to understand the capacity of fishes to tolerate warming.

A primary methodology used to evaluate upper thermal limits in fishes is the critical thermal maximum (CT_max_) protocol (Lutterschmidt and Hutchison, 1997). This involves warming a fish progressively in steps and uses loss of equilibrium (LOE) as the tolerance endpoint, the temperature at which the fish loses its ability to maintain orientation and turns over. Although the CT_max_ does not cause mortality, LOE is an incipient lethal threshold because, in the wild, the animal would be unable to escape from the thermal conditions and so its survival would be threatened (Lutterschmidt and Hutchison, 1997). It has also proven rather difficult to understand what causes LOE at CT_max_ in fishes (Ern et al., 2016, 2017; Jutfelt et al., 2019; Wang et al., 2014). It is essential to understand the physiological mechanisms that underlie tolerance of warming because this will greatly improve the ability to model and project the effects of global warming on fish populations and communities (Jørgensen et al., 2012; McKenzie et al., 2016).

The dominant hypotheses for the physiological mechanism that underlies tolerance of warming in fishes are the Fry paradigm (Fry, 1947, 1957, 1971) and its mechanistic elaboration, the oxygen and capacity limited thermal tolerance (OCLTT) hypothesis (Pörtner, 2010; Pörtner et al., 2017). The core concept in the Fry paradigm and the OCLTT hypothesis is that thermal tolerance is limited by the capacity of the cardiorespiratory system to meet tissue oxygen demands (Fry, 1971; Pörtner, 2010; Pörtner and Farrell, 2008). That is, when fishes are warmed, their basal metabolism is progressively accelerated until, at a certain warm temperature, the oxygen demands for simple maintenance require the entire cardiorespiratory capacity for oxygen supply. At that temperature, the aerobic scope (AS) for vital activities beyond maintenance (e.g. exercise, growth and reproduction) is zero; exceeding that temperature threshold rapidly leads to overall physiological collapse (Eliason et al., 2011; Pörtner, 2010; Pörtner and Farrell, 2008; Schulte, 2015). These temperature thresholds are expected to be species-specific and depend upon the species' ecology and evolutionary history. The mechanism behind the OCLTT is proposed to be universal (Fry, 1971; Pörtner, 2010) but this is currently an active debate (Farrell, 2016; Jutfelt et al., 2018; Lefevre, 2016; Schulte, 2015), in particular because there is evidence that LOE at CT_max_ may not be linked to limitations in tissue oxygen supply (Ern et al., 2016, 2017; Wang et al., 2014).

As such, there is a need for alternative protocols to evaluate sub-lethal sensitivity to acute warming in fishes, especially which use ecologically relevant endpoints that are less extreme than LOE and which are more amenable to understanding their underlying physiological mechanisms. If limitations to tissue oxygen supply do underlie a collapse in performance during warming, then this could be tested by imposing a level of aerobic metabolic work upon a fish and measuring their capacity to sustain it while their overall tissue oxygen demands are being accelerated by progressive increases in temperature. When sockeye salmon, *Oncorhynchus nerka*, were exposed to progressive warming while they were swimming at a high sustained aerobic speed in a swim tunnel, at a certain warm temperature they exhibited signs of unsteady anaerobic swimming and then fatigued (Steinhausen et al., 2008).

We therefore investigated the hypothesis that fatigue from sustained aerobic swimming provides a sub-lethal threshold for tolerance of acute warming in fishes, which is more sensitive (occurs at a lower temperature) than LOE at CT_max_. We studied two widespread tropical freshwater species, a cichlid that originates from Africa, the Nile tilapia, *Oreochromis niloticus* (Linnaeus 1758), and a serrasalmid from South America, the pacu, *Piaractus mesopotamicus* (Holmberg 1887). For each individual fish, we first performed an incremental swim test to reveal its gait transition speed (*U*_GT_), where it transitioned from steady aerobic to unsteady anaerobic swimming (Marras et al., 2013). After suitable recovery, we set each fish to swim at 85% of their own *U*_GT_ and warmed them in steps, to identify the temperature at which they fatigued, which we denoted as CT_swim_. We then compared CT_swim_ of all fishes with their own CT_max_, the temperature of LOE when warmed at the same rate under static conditions. We also expected to gain qualitative evidence that the collapse in performance at CT_swim_ was linked to an inability to meet tissue oxygen demand, in particular that fatigue was preceded by a gait transition from steady aerobic to unsteady anaerobic swimming. This would indicate that the CT_swim_ protocol could be useful in testing the expectations of the OCLTT paradigm. Finally, we measured rates of oxygen uptake during the initial swim test and during the CT_swim_, to gain insight into how acute warming influenced overall capacity for oxygen supply, relative to capacity at the initial acclimation temperature.

## MATERIALS AND METHODS

### Ethical approval

The experiments were performed according to the regulations of the Brazilian National Council for Control of Animal Experimentation (CONCEA). The protocol was approved by the Ethics Committee on Animal Use of the Federal University of São Carlos (CEUA/UFSCAR), protocol number CEUA 3927151016.

### Animals

Juvenile tilapia and pacu of both sexes were provided by Piscicultura Polettini in Mogi Mirim (São Paulo state) and transported to the Department of Physiological Sciences, Federal University of São Carlos (São Carlos, SP). There, each species was held in a 500 liter holding tank supplied with well-aerated water at 25±1°C from a recirculating biofilter system, and fed *ad libitum* every second day with commercial pellets. After a period of at least 2 weeks, animals were anesthetized (0.1 g l^−1^ benzocaine) and tagged (PIT) for individual identification. They were allowed to recover in routine holding conditions for at least 1 week before use in the experiments.

### Swimming performance and metabolic phenotype at acclimation temperature

Each fish was measured for mass (to the nearest 0.1 g) and for fork length, height and width (to the nearest mm), to calculate the solid blocking effect in the swim flume, and to measure relative swimming speed in body lengths (BL) s^−1^ (Bell and Terhune, 1970). Swimming respirometry was performed with a Steffensen-type swim tunnel respirometer constructed of Plexiglas (volume 13.4 liters), designed to exercise fish in a non-turbulent water flow with a uniform velocity profile (McKenzie et al., 2007, 2012).

Each fish was placed in the respirometer and allowed to recover overnight, swimming at a gentle current speed equivalent to 1 BL s^−1^. The respirometer was supplied with a constant flow of aerated biofiltered water at 26°C. The next morning the fish was exposed to progressive increments in swimming speed of 1 BL s^−1^ every 30 min until 5 BL s^−1^ was reached. Beyond this, it was exposed to constant acceleration at 0.1 BL s^−1^ every 10 s until fatigue, with this speed noted as maximum swimming speed (*U*_max_; Marras et al., 2013). During the constant acceleration element of the protocol, *U*_GT_ was when fish began to engage an unsteady anaerobic gait, rapidly leading to fatigue (Marras et al., 2013). The aerobic gait comprised steady body-caudal swimming (rhythmic beating of the tail) that relies upon slow twitch oxidative muscle. The unsteady anaerobic gait comprised intermittent powerful tailbeats that propelled the fish to the front of the swim tunnel, after which it coasted to the back, before repeating the tailbeat, so-called ‘burst and coast’ swimming that relies on recruitment of fast-twitch glycolytic muscle (Marras et al., 2013; Webb, 1998). When a fish fatigued and fell back against the rear screen of the swim tunnel, speed was immediately reduced to 1 BL s^−1^ and it was allowed to recover for 30 min. The fish was then removed from the tunnel and placed in its holding tank to recover for at least 96 h before any subsequent protocol.

Measurements of O_2_ uptake (*Ṁ*_O_2__; mmol kg^−1^ h^−1^) were made at each swimming speed between 1 and 5 BL s^−1^, by intermittent stopped-flow respirometry (Steffensen, 1989) over a 15 min cycle, providing two measures of *Ṁ*_O_2__ for each speed. Water oxygen concentration was recorded continuously using an optical oxygen probe and meter (Fibox, Pre-sens GmbH, Regensburg, Germany, www.presens.de) and associated software (Pre-sens Oxyview). The *Ṁ*_O_2__ was then calculated considering the rate of decline in oxygen concentration, the water volume in the swim tunnel and the mass of the fish (McKenzie et al., 2003, 2007). For each individual fish and swim test, a least-squares exponential regression was applied to the relationship between swimming speed and the *Ṁ*_O_2__ measurements. Extrapolation back to the *y*-intercept, a notional swimming speed of zero, was employed to correct for the contribution to *Ṁ*_O_2__ of locomotor muscle activity (Brett, 1964; Fry, 1971). This was considered an estimate of standard metabolic rate (SMR). Active metabolic rate (AMR) was also calculated based on the exponential regression, by resolving *Ṁ*_O_2__ at the maximum aerobic swimming speed (*U*_GT_). Aerobic scope (AS) was calculated as AMR minus SMR.

### CT_max_ for swimming (CT_swim_) protocol

Control trials were run to establish that fishes could swim uninterrupted for at least 9 h at 85% of their own *U*_GT_, at their acclimation temperature of 26°C, which exceeded the duration of the CT_swim_ protocol. Measurements of *Ṁ*_O_2__ were collected for 10 min once every 30 min throughout, using the same methods as described for swimming respirometry above.

Fishes were then challenged to swim at 85% of their pre-determined *U*_GT_ and exposed to stepwise warming at a rate of 1°C every 30 min. The response variable in CT_swim_ was fatigue, the temperature at which the fish refused to swim and fell back against the rear screen. CT_swim_ was calculated as the last temperature step completed plus the proportion of the last temperature step that the fish tolerated prior to fatigue. Immediately upon fatigue the fish was placed into a recovery tank containing water at 26°C. Once they had regained normal swimming behavior they were moved to the holding tanks to recover for at least 96 h prior to any subsequent protocol. Measurements of *Ṁ*_O_2__ were made over the last 10 min at each temperature step. The highest *Ṁ*_O_2__ measurement was identified for each fish and named *Ṁ*_O_2_max_–CT_swim_.

### Static CT_max_ protocol

Four individuals from each species were selected randomly and placed overnight in a 68 liter tank containing aerated water at 26°C, to recover from handling. The following morning, the water in the tank was warmed in steps of 1°C every 30 min. The tank was well aerated to maintain dissolved oxygen levels and avoid thermal stratification. Loss of equilibrium (LOE) was used as the indicator of thermal tolerance, involving complete loss of dorsoventral orientation. CT_max_ was then recorded as the last temperature step fully completed plus the proportion of the last step that the fish endured prior to LOE. Upon LOE, each fish was rapidly transferred to a 68 liter recovery tank containing water at 26°C. After at least 30 min and when they had recovered normal swimming behavior, they were returned to their holding tank to recover for at least 96 h prior to further experimentation.

### Statistical analysis

All statistics and models were produced using R v. 3.4.0 (http://www.R-project.org/) using the function lmer in package lme4 (https://CRAN.R-project.org/package=lme4). Metabolic and performance variables were compared between species by *t*-test and within species by paired *t*-test, with *P*<0.05 taken as the limit for significance. Critical thermal (CT) values were examined using a general linear model with CT as the response variable, and species and protocol (CT_max_ versus CT_swim_) as responses variables. An interaction between species and protocol was initially included but was non-significant, and so was removed and the model re-run. Linear mixed-effects (LME) models were constructed to examine the effect of warming on oxygen uptake during swimming, with oxygen uptake as the response variable, species as a categorical explanatory variable, treatment (warming versus constant 26°C) as a categorical explanatory variable, temperature as a continuous explanatory variable, and fish ID as a random effect. The full model was first fitted using restricted maximum likelihood (REML) estimation to compare possible random structures by likelihood ratio testing. We compared the random intercept model with fish identity nested within group as a random factor with random slope models where the slope estimates were allowed to vary among individuals for oxygen availability. Random slopes did not improve model parsimony and so only random intercepts were used for fish ID nested within group. Two-way interactions among species, treatment and temperature were initially included in the full model but removed when non-significant, and the models re-run. Model assumptions of linearity, normality and homogeneity of residuals were confirmed by inspecting plots of model residuals versus fitted values. Model *r*^2^ values were computed using the MuMIn 1.9.13 package for R (https://CRAN.R-project.org/package=MuMIn). For LME models, this included marginal *r*^2^ and conditional *r*^2^, which indicate the variance explained by fixed factors, and by both fixed and random factors, respectively (Nakagawa and Schielzeth, 2010). Data used for the analyses have been deposited in Dryad (doi:10.5061/dryad.cjsxksn2v).

## RESULTS

### Swimming performance and metabolic phenotype at acclimation temperature

The two species showed a significant difference in their swimming performance; mean *U*_GT_ and *U*_MAX_ were higher in pacu than in tilapia (Table 1). Mean SMR was lower in pacu than in tilapia, but there were no differences in AMR or AS (Table 1).

**Table 1.**
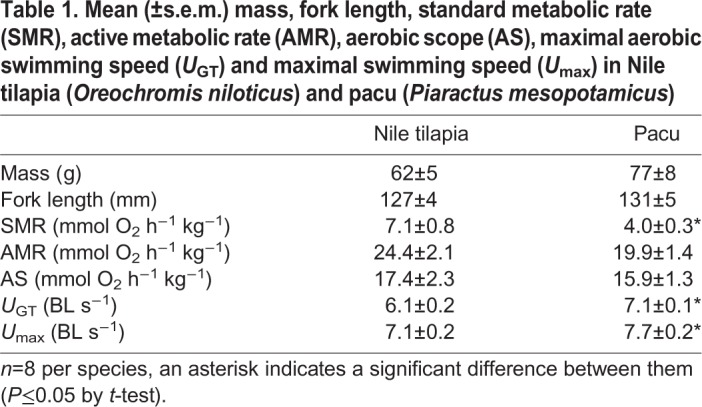
**Mean (±s.e.m.) mass, fork length, standard metabolic rate (SMR), active metabolic rate (AMR), aerobic scope (AS), maximal aerobic swimming speed (*U*_GT_) and maximal swimming speed (*U*_max_) in Nile tilapia (*Oreochromis niloticus*) and pacu (*Piaractus mesopotamicus*)**

### Tolerance of acute warming: CT_swim_ versus CT_max_

**Fig. 1.**
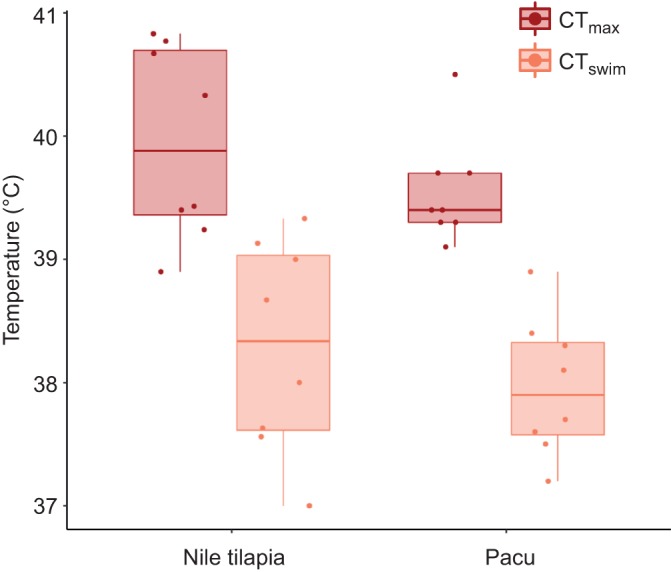
**Box plot of data for CT_swim_ (orange data) or CT_max_ (red data) in two fish species, Nile tilapia (left) and pacu (right).** Boxplot lower and upper hinges represent the 25th and 75th percentiles, respectively; the horizontal line within the box is the median; the length of whiskers represents the range of data points between each hinge and 1.5× the difference between the 25th and 75th percentiles. Data beyond these limits are outliers. Each point is one fish, *n*=8 in all cases.

At their acclimation temperature, all fishes swam using a steady aerobic gait for at least 9 h at 85% of their *U*_GT_ without any sign whatsoever of fatigue. During the CT_swim_ protocol, all individual fishes swam using the steady aerobic gait until a certain warm temperature, at which they started to engage unsteady burst and coast anaerobic swimming, which was followed by fatigue with the fish falling back against the rear screen or simply refusing to swim. The gait transition was qualitatively indistinguishable from that observed during the constant acceleration test. No fish lost equilibrium during the CT_swim_ protocol and, in both species, the CT_swim_ was significantly lower than CT_max_, by almost 2°C (Table 2, Fig. 1). During CT_max_, fish showed signs of erratic behavior and sideways rolling prior to complete LOE. Neither of the warming protocols caused any mortality and all fish recovered normal behavior within 30 min of returning to water at 26°C. There were no differences in mean CT_swim_ or CT_max_ between the two species (Table 2, Fig. 1).

**Table 2.**
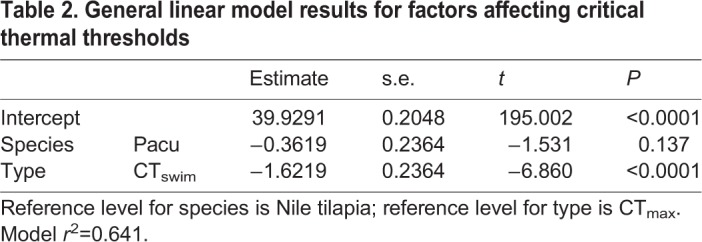
**General linear model results for factors affecting critical thermal thresholds**

### Effects of acute warming on metabolic rate

**Fig. 2.**
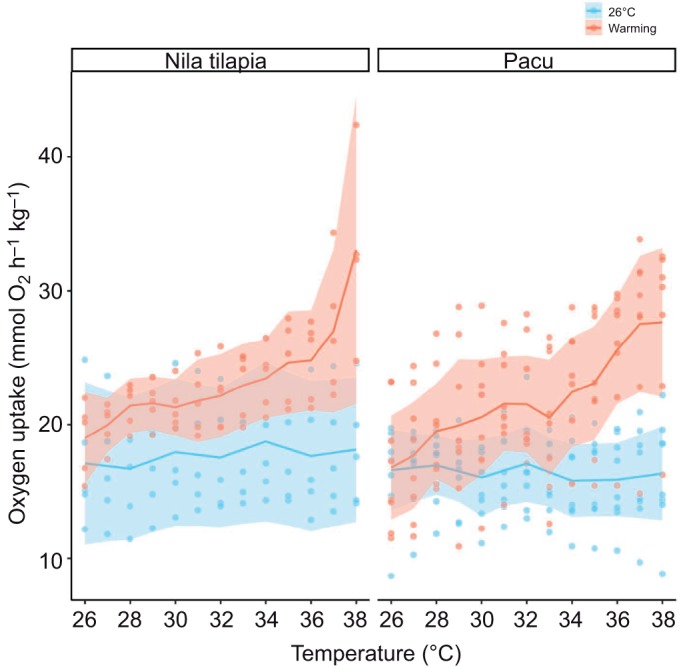
**Oxygen uptake during the CT_swim_ protocol, when Nile tilapia (left) or pacu (right) were swimming aerobically and warmed 1°C every 30 min, from their acclimation temperature of 26°C (red data and curve), or when the same fishes swam aerobically for the same period at their acclimation temperature (blue data and curve).** Swimming speed in both cases was set at 85% of each individual's gait transition speed in an incremental swim trial (see Materials and Methods for more details). The shaded area is the 95% confidence interval at each temperature. Each point is a fish measured at that temperature, or that interval for the control swim at 26°C.

During the control swim, there was no change in *Ṁ*_O_2__ for 9 h in either species (Fig. 2), and their mean *Ṁ*_O_2__ (Table 3) was approximately 75% of their mean AMR (Table 1). The warming during CT_swim_ was associated with a profound increase in *Ṁ*_O_2__ for both species (Fig. 1, Table 4). The mean *Ṁ*_O_2_max_ (Table 3) was significantly higher than each species' mean AMR at their acclimation temperature (Table 1). The species did not differ in their *Ṁ*_O_2__ at 26°C at the outset of the CT_swim_, or in their *Ṁ*_O_2_max_. As a result, the aerobic scope due to acute warming was similar in both species (Table 3). These species differences were reflected in the species effect in the LME models (Table 4).

**Table 3.**
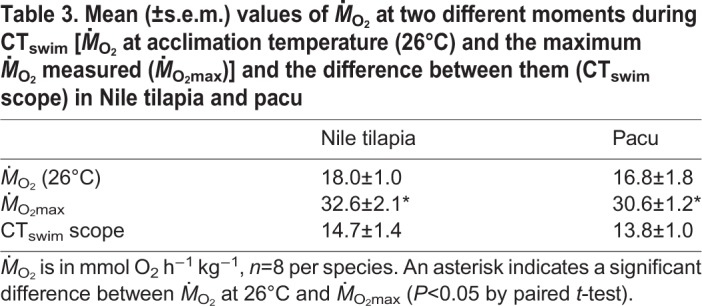
**Mean (±s.e.m.) values of *Ṁ*_O_2__ at two different moments during CT_swim_ [*Ṁ*_O_2__ at acclimation temperature (26°C) and the maximum *Ṁ*_O_2__ measured (*Ṁ*_O_2_max_)] and the difference between them (CT_swim_ scope) in Nile tilapia and pacu**

**Table 4.**
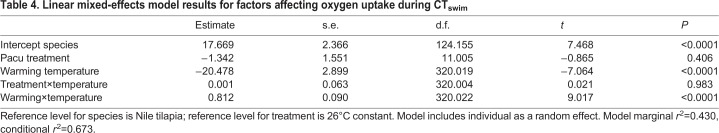
**Linear mixed-effects model results for factors affecting oxygen uptake during CT_swim_**

## DISCUSSION

The results demonstrate that, in two fish species, CT_swim_ can be used to define tolerance of acute warming with fatigue as a sub-lethal endpoint rather than the more extreme incipient lethal endpoint of LOE at CT_max_ (Mauduit et al., 2016; Rezende et al., 2014). The results indicate that, if exposed to acute warming events in the wild, the ability of fishes to perform aerobic metabolic work would be constrained at temperatures significantly below those at which their survival was directly threatened, which has clear ecological implications. It is also true that the likelihood of these two species experiencing extreme warming events up to 38°C, the approximate temperature of their CT_swim_, is much higher than for events up to 40°C, the temperature of their CT_max_ (Wong et al., 2014; Collins et al., 2019; Frölicher and Lauffkötter, 2018). This protocol needs to be extended to more fish species, but the present study establishes that CT_swim_ can provide an ecologically relevant endpoint that is significantly more sensitive than CT_max_.

In addition to being more sensitive than the CT_max_, the CT_swim_ protocol is interesting because the underlying mechanisms for fatigue seem clearly related to capacity for oxygen supply. Various studies have demonstrated a disconnect between CT_max_ and capacity for oxygen supply; whether CT_max_ is oxygen-dependent appears to vary with species. The physiological mechanisms of oxygen-independent CT_max_ are not understood and may be linked to effects of acute warming on membrane integrity and nerve function. These points have all been raised to argue against the universality of the OCLTT framework (Ern et al., 2014, 2016, 2017; Jutfelt et al., 2019; Wang et al., 2014). By contrast, the CT_swim_ protocol specifically targets metabolic constraints caused by warming. The control swimming at 85% of *U*_GT_ proved that both fish species could perform sustained aerobic work at a constant metabolic rate for very extended periods, with no signs whatsoever of fatigue. Therefore, fatigue during the CT_swim_ was not due to the metabolic work of swimming but, rather, to the metabolic load imposed by warming. It is very interesting that, at a high temperature close to their CT_swim_, all individuals of both species began to engage unsteady burst and coast swimming in order to maintain station in the water current, which rapidly led to fatigue. This response was qualitatively indistinguishable from the gait transition observed during an incremental swim challenge, which indicates that it was due to a similar underlying mechanism.

The teleost heart is an aerobic organ and obtains much of its oxygen from venous return in the single circulation, blood that has unloaded much of its oxygen to respiring tissues (Farrell and Jones, 1992; Jones and Randall, 1978). It has been suggested that during an incremental swim challenge, fishes engage the gait transition towards anaerobic swimming when oxygen levels in venous blood returning to the heart drop below a critical level. By engaging the white muscle, the fish reduces rates of oxygen extraction by the working red muscle and so venous oxygen supply to the heart is assured (Farrell and Clutterham, 2003; McKenzie and Claireaux, 2010; McKenzie et al., 2004). The observed gait transition prior to fatigue during the CT_swim_ may suggest a similar decline in venous oxygen levels, which risked compromising oxygen supply to the working heart (McKenzie and Claireaux, 2010). That is, the cardiorespiratory system could no longer meet the combined oxygen demands of thermal acceleration of metabolism and sustained aerobic exercise. Steinhausen et al. (2008) found that fatigue from exercise in the chinook salmon during acute warming coincided with cardiac pumping capacity reaching its maximum. There is other evidence that the capacity of the heart to maintain tissue oxygen supply may be a critical element of tolerance of acute and chronic warming in fishes (Clark et al., 2008; Sandblom et al., 2016), so this is an interesting issue for future research with the CT_swim_ protocol.

The methodology to properly explore the Fry paradigm and the OCLTT hypothesis is to measure SMR and AMR in fish held at a range of temperatures, to construct an AS performance curve. This is very time-consuming and very difficult to perform on a single individual (Claireaux et al., 2006; Claireaux and Lefrançois, 2007; Eliason et al., 2011), which seriously constrains exploration of the universality of the mechanisms across species, and exploration of questions regarding individual variation and phenotypic plasticity within species. Although we cannot be absolutely certain of the mechanism that led to fatigue in the CT_swim_ test, the evidence clearly indicates that it was linked to problems with meeting metabolic oxygen demand. This is consistent with the OCLTT hypothesis. Therefore, the CT_swim_ can be used directly to explore the mechanisms underlying warming-induced fatigue, to confirm that they do reflect constraints on meeting metabolic oxygen demand, and to establish whether the response is universal across fish species. The CT_swim_ protocol could also be used to explore questions regarding plasticity and resilience of fish species to climate change and thermal stress. The rate of environmental change associated with global warming is too fast to be coped with by adaptive evolutionary responses and, therefore, the resilience of species will depend upon migration, the existence of tolerant genotypes, and phenotypic plasticity (Bell, 2013; Gonzalez and Bell, 2013). The CT_swim_ protocol is ideal for evaluating individual variation in thermal sensitivity, based upon oxygen supply capacity, and the potential for phenotypic plasticity to offset any constraints.

It is interesting that the *Ṁ*_O_2_max_ was very significantly higher than the AMR of each species at their acclimation temperature. The exponential increase in *Ṁ*_O_2__ during the CT_swim_ was purely due to the effects of temperature on metabolism, as *Ṁ*_O_2__ did not change when fishes swam at 26°C for 9 h. This shows that acute warming increases the cardiorespiratory capacity of these two tropical species, in an immediate and rapid manner. Claireaux et al. (2006) measured *U*_GT_ (called *U*_max_ in that study) and associated respiratory metabolism in European seabass, *Dicentrarchus labrax*, that were seasonally acclimatized to temperatures between 7 and 30°C. They found that warming was associated with an increased capacity for aerobic metabolism, energy flux and metabolic scope. The current data indicate that these effects can also be seen during acute warming. When Steinhausen et al. (2008) exposed chinook salmon to acute warming when swimming at 75% of their critical swimming speed (*U*_crit_), the thermal effects on oxygen uptake were much less pronounced than the exponential increase in metabolic rate observed in Nile tilapia and pacu. Salmonids are considered rather stenothermic species (Eliason et al., 2011). The remarkable cardiorespiratory plasticity in response to acute warming by these two tropical species may reflect an evolutionary history of exposure to warm and changeable water temperatures. The mechanisms underlying this plasticity remain to be explored, but a release of catecholamines and increased performance of the cardiac pump may be of major importance (Jayasundara and Somero, 2013; Nyboer and Chapman, 2018).

In conclusion, fatigue in a CT_swim_ protocol provides a sub-lethal threshold for tolerance of warming that is relevant because of the ecological importance of swimming for fishes and that is more sensitive to potential extreme events than LOE in a CT_max_. It also has the advantage that the underlying mechanism for fatigue in CT_swim_ appears to be linked to constraints on tissue oxygen supply, such that this can now be explicitly investigated. The protocol has applications in testing hypotheses such as the OCLTT (Jutfelt et al., 2018; Pörtner, 2010) and gill oxygen limitation (Lefevre et al., 2017, 2018; Pauly and Cheung, 2017), or in exploring how and why individuals, populations and species might differ in their sub-lethal tolerance of warming (Anttila et al., 2013; Fangue et al., 2009; Mauduit et al., 2016; Roze et al., 2013; Schulte et al., 2011).
